# Epidemics and policy: the dismal trade-offs

**DOI:** 10.1007/s40888-022-00279-3

**Published:** 2022-07-18

**Authors:** Francesco Flaviano Russo

**Affiliations:** University of Naples Federico II and CSEF, Via Cintia, Monte Sant’ Angelo, 80126 Naples, Italy

**Keywords:** Lockdown, Testing, Pathogen, Quarantine, E1, I1, H12

## Abstract

I propose a stochastic SIR-Macro model to study the effects of alternative mitigation policies to cope with an epidemic. Lockdowns that force firms to close and that discontinue social activities slow down the progression of the epidemic at the cost of reducing GDP and increasing debt and, on average, decrease mortality. Testing-Tracing-Quarantine policies decrease mortality at a lower cost, but they are effective only if thorough. I find that lockdowns work best in case of a bigger average family size, of a diffused labor market participation and of a bigger average firm size.

## Introduction

The COVID-19^1^ pandemic will most likely be the defining event of this century: an astonishing number of people died, while the policies implemented to cope with it resulted in major recessions and in a surge of public debt, with potentially severe long-run effects. However it has not been the only large-scale infectious disease event in recent history. Even abstracting from influenza, a recurrent winter event, although not as deadly, and from AIDS, still ravaging, there are at least four others worth mentioning: the first SARS, the Swine Flu, the MERS and Ebola.

Faced with a rapidly spreading contagious disease, epidemiologists typically prescribe social distancing, especially if asymptomatic infections are possible, mainly to ease the pressure on hospitals, which might not have enough resources to simultaneously treat all of the infected. Social distancing can be achieved in several ways: shutting down schools, universities, offices, plants, restaurants, bars and shops; closing the national, regional of even the city borders; forcing everyone to stay home; in the extreme limit, confining all of the infected away, alongside those who had contacts with them, as in Saramago’s “Blindness”.

Lockdowns are obviously extremely costly. The GDP will plummet if productive activities are closed, leaving many workers without income and many entrepreneurs without cash-flow. Governments typically support both workers and entrepreneurs with transfers, but such deficit-financed policies increase debt and, for the already highly indebted countries, this translates into worse borrowing terms and into a higher probability to implement restrictive fiscal policies in the near future. Moreover, some firms will be forced to shut down even in case of financial aid, and the older, among the unemployed, will no longer be able to find another job.

Lockdowns are not the only policy alternative. First, because there is the possibility to let the epidemic unfold without any intervention, to quickly reach herd immunity. A further alternative is the so-called Testing-Tracing-Quarantine (TTQ) policy, which entails identifying and testing all of those who came into contact with the already discovered infected individuals.

The main goal of this work is to study the effects of alternative mitigation policies deigned to cope with an epidemic, conditional on the pathogen characteristics and on country-level characteristics. I build a model that nests the structure of a stochastic Susceptible-Infected-Recovered (SIR) model (Allen, 2017; Kermack and McKendrick, 1927) within an equilibrium dynamic economic model with heterogeneous agents and firms. Its key feature is that the evolution path of the epidemic, for given policy, is contingent on the identity of the infected individuals, with a resulting variability of potential outcomes that depends on who gets the pathogen at each stage. Moreover, the rich model structure allows to study the design of the mitigation policies in great detail.

When comparing policies, I focus on what I define the *dismal trade-offs*, respectively between lives saved and output lost and between lives saved and debt. I propose two measures to evaluate them: the Output Dismal Ratio (ODR), defined as the average percentage of the population spared for each percentage point of output lost, and the Debt Dismal Ratio (DDR), defined as the average percentage of the population spared for each additional percentage point of public debt.

I find that, on average, TTQ policies work best, in the sense that they yield the lowest dismal ratios, although both lockdowns and TTQ policies do not always result in positive ODR and DDR. The gains from both policies are higher in case of more aggressive and more deadly diseases, and at intermediate levels of the treatment capacity of the health system. Moreover, lockdowns are more effective in case of smaller families, bigger firms, denser societies and in case of a more diffused labor market participation. Conversely, TTQ policies, to work properly, must be able to isolate a big fraction of the asymptomatics. In terms of mitigation policy design, I find that lockdowns must be prolonged until the pathogen effective reproduction number (the number of new infections caused by each infected individual) drops below a very small value, and that severe lockdowns, that close a large number of economic activities, are not appropriate if the average firm size is small. Moreover, social-only lockdowns are less costly from a macroeconomic standpoint, but they might be insufficient in case of big average family and firm size. In a two-regions extension of the model, I also show that closing the borders to travel is always beneficial.

The rest of the paper is organized as follows. I briefly discuss the related literature in Sect. 2. Section 3 contains the model description, while, in Sect. 4, I fully explain the calibration and simulation details. Section 5 summarizes the main simulation results. In Sect. 6, I explore the robustness of the results. In Sect. 7, I discuss several model extensions. Section 8 concludes. An appendix contains additional results and a brief description of the model solution algorithm.

## Related literature

The literature on the economic effects of the epidemics sprouted as soon as many governments implemented lockdowns to contrast the COVID-19 pandemic at the beginning of 2020. Examples include, among others, Acemoglu et al. (2021; Atkeson (2020); Alvarez et al. (2021); Collard et al. (2020); Eichenbaum et al. (2020a, 2020b); Glover et al. (2020); Kaplan et al. (2020 and Piguillem and Shi (2022). The main contribution of this work is to study the factors that influence the performance of the mitigation policies, and to highlight their variability of outcomes.

Berger et al. (2022); Eichenbaum et al. (2020b; Piguillem and Shi (2022) show that TTQ policies are effective at smoothing the peak of the infection and at reducing both the mortality and the economic impact of the epidemic. My results are in line with their analysis.

In Bethune and Korinek (2020); Chang and Velasco (2020); Collard et al. (2020); Eichenbaum et al. (2020a); Farboodi et al. (2021); Garibaldi et al. (2020); Jones et al. (2021); Krueger et al. (2022); Piguillem and Shi (2022) and Toxvaerd (2020), the agents react to the epidemic with individual actions that limit the spread of the pathogen, such as decreasing consumption, labor supply and social interactions, reallocating consumption away from goods consumed socially, or wearing protective devices. The dynamic evolution of the epidemic is therefore endogenous in their models, and the optimal policy intervention milder. I abstract from such endogenous reactions.

Kaplan et al. (2020) focus on the heterogeneous consequences of the pandemic by income, wealth and sector of activity, showing that policy interventions are distributional choices. I abstract from these heterogeneities, but I focus on family and firms heterogeneities. Acemoglu et al. (2021); Favero et al. (2020); Glover et al. (2020) build models with heterogeneous health risks by age and/or sector of activity to show the effects of fine-tuned public policies contingent on specific individual characteristics. My framework, although more simplified, also allows for such an analysis.

Similarly to Collard et al. (2020) and Favero (2020), I focus on the trade-off between mortality and output loss, extending the discussion to public debt. This approach is different from, among others, Alvarez et al. (2021); Eichenbaum et al. (2020a); Glover et al. (2020) and Hall et al. (2020), who look instead for the optimal policy contingent on a monetized value of life.

Few works studied the economic consequences of epidemics, and of diseases in general, before the COVID-19 2020 outbreak. Young (2005) studies the long-run impact of the HIV epidemic in South Africa; Goenka et al. (2014) the long-run consequences of epidemics on economic growth; Greenwood et al. (2019) the potential effects of alternative policies to stem the HIV diffusion in Malawi.

## The model

I consider a closed economy where a total of *N* agents live and work before an epidemic strikes. An agent *i* is represented by the vector $x_{i j t}$, where *t* is the time period and where *j* indexes the firm where she works. The agents who do not work, with $j=0$, are either pensioners, unemployed or underage. The vector *x* is composed of five binary elements that describe the individual status towards the infection. Specifically, $x_{i j t}= \{ f_{i j t},a_{i j t},s_{i j t},u_{i j t},d_{i j t} \}$, where $f_{i j t}=1$ in case of infection with symptoms, $a_{i j t}=1$ in case of infection without symptoms, $s_{i j t}=1$ in case of susceptibility to infection, $u_{i j t}=1$ in case of immunity upon recovery, and $d_{i j t}=1$ in case of death. I assume that all agents are susceptible in $t=0$, as it is the case for new pathogens. Moreover, I do not model demographics independently from the epidemic, so all deaths are due to the pathogen and there are no new entries in the labor market. The stock of infected agents with symptoms is $F_{t}=\sum _{i=1}^{N} f_{i j t}$, the stock of infected without symptoms $A_{t}=\sum _{i=1}^{N} a_{i j t}$, the stock of susceptibles $S_{t}=\sum _{i=1}^{N} s_{i j t}$, the stock of immunes $U_{t}=\sum _{i=1}^{N} u_{i j t}$ and total deaths $D_{t}=\sum _{i=1}^{N} d_{i j t}$.

### Families and firms

The agents belong to *I* families, represented by the following collection of sets $\{n_{1}, n_{2} \dots n_{I}\}$. The number of members of each family $h \in \{1,2,\dots I \}$ is equal to the cardinality of each set $n_{h}$. Thus: $\sum _{h=1}^{I} \vert n_{h} \vert = N$. The agents are associated to their respective families by the function $N(i)=n_{h}$ if $i \in n_{h}$.

There is a total of *J* firms in the economy, modeled by the collection of sets $\{m_{1}, m_{2} \dots m_{J}\}$. Firms have ${\hat{M}}_{j}=\vert m_{j} \vert$ employees at full capacity, where ${\hat{M}}_{j}=1$ stands for self-employed agents. The total number of employed agents at full capacity is $\sum _{j=1}^{J} {\hat{M}}_{j} = L < N$. The firms employ the workers, which are all equally productive, to produce the unique undifferentiated product of this economy. I assume that the workers are assigned to the firms randomly and that members of the same family can work together. This random assignment is an important source of variation in policy outcomes: if agents from big families are assigned to big firms, the pathogen will spread faster.

Workers are paid the fixed wage *w*. The pensioners and the unemployed are paid a transfer $\theta w$, with $\theta \le 1$. For simplicity, I assume that all families receive the same amount $\theta w$ for each underage member. The product is sold at the market clearing price $p_{t}$ and the firms are price takers. I assume that symptomatic agents do not go to work. Asymptomatic agents work only if quarantined after a positive test result. I assume that it is not possible for the firms to replace the sick or quarantined workers with the unemployed.

The firm is forced to temporarily close if the number of non-sick workers drops below a lower bound ${\bar{M}}_{j}$. A firm that produces zero output in a given period reopens the next with time-independent probability $\lambda$. This assumption adds hysteresis to the model dynamic. For simplicity, I assume that $\lambda$ is not a function of the number of periods in which the firm has been closed while, in reality, it is a decreasing function of it. Moreover, I will not explicitly study the effects of policies that increase $\lambda$, such as liquidity provisions or public guarantees on private loans. The individual production function of firm *j* is therefore:

1 $$\begin{aligned}  { y_{j,t}={\mathbbm {1}}_{ [M_{j t}  > \, {\bar{M}}_{j}] } \left[ \Lambda +(1-\Lambda ) {\mathbbm {1}}_{ [M_{j t-1} \ge \, {\bar{M}}_{j}] } \right] \, M_{j t}^{\alpha } } \end{aligned}$$

where ${\mathbbm {1}}$ is the indicator function, $\Lambda$ is a draw from Bernoulli random variable with parameter $\lambda$. $M_{j t}$ is the number of available workers for firm *j* in period *t*, either because they are susceptible, immunes or asymptomatic and not quarantined:

2 $$\begin{aligned} { M_{j t}={\hat{M}}_{j t}-\sum _{j\in m_{j}} f_{ij t}-\sum _{j\in m_{j}} \kappa _{i t} \, a_{i j t} } \end{aligned}$$

with $k_{i t}=1$ in case the asymptomatic worker *i* is quarantined in period *t* after a positive test result. Total production is simply the sum of all outputs from the *J* firms, while the potential output of the economy is the sum of all outputs at full capacity: $Y_{t}=\sum _{j=1}^{J} y_{j t} \quad \le \quad {\hat{Y}}=\sum _{j=1}^{J} {\hat{M}}_{j}^{\alpha }$. I use the output gap $(Y_{t}-{\hat{Y}})/{\hat{Y}}$ as a measure of the recessionary effect of the epidemic and of the mitigation policies. Profits, if positive, are taxed at rate $\tau ^{y}$, and there are no tax credits in case they are negative. I also assume that profits are not distributed as dividends.

Agents choose consumption optimally given the expected future earnings. Infected agents who cannot work (symptomatics or asymptomatics and quarantined) earn the same amount of the transfer $\theta w$. The planning horizon is limited to the duration of the epidemic, that ends at time *T*, expectations are rational and there is perfect foresight. The agents are allowed to save and borrow at the exogenous interest rate *r*. The optimization problem of the consumers is therefore:

3 $$\begin{aligned} { \max _{\{c_{i j t+s}\}_{s=0}^{T-t}} \, E_{t} \sum _{s=0}^{T-t} \beta ^{s} \upsilon (c_{i j t+s}) } \end{aligned}$$

subject to the (consolidated) present value budget constraint:

4 $$\begin{aligned} { E_{t} \sum _{s=0}^{T-t} \frac{1}{(1+r)^s} p_{t+s} \, c_{i j t+s} = w \, (1-\tau ^{w}) \, E_{t} \sum _{s=0}^{T-t} \frac{1}{(1+r)^s} \left[ \theta f_{i j t+s}+(1-\kappa _{i t}+\kappa _{i t} \theta )a_{i j t+s} +u_{i j t+s} \right] } \end{aligned}$$

where $\tau ^{w}$ is the tax rate on wages and transfers.

In the model there is no endogenous response of the labor supply to the spread of the disease. This is equivalent to assuming that the agents do not have enough savings, or borrowing ability, to afford a labor supply reduction. Moreover, agents do not reduce their consumption when faced with a high risk of contracting the pathogen, say at the supermarket or at a corner store. This assumptions keep the model tractable, but they are restrictive. However the alternative model with an endogenous reduction of consumption and of the labor force participation proportional to the spread of the pathogen, namely with an endogenous mitigation mechanism, implies lower overall gains from the implementation of lockdowns or testing policies. In such a setting, the individual responses might be enough to stem the pathogen diffusion, thereby limiting its impact on the economy. Therefore the results from my policy counterfactuals, when compared to the no-policy benchmark, must be interpreted as upper bounds to what could be achieved with mitigation policies. A further issue is that the individual responses could be contingent on the implemented policies, for instance they could be stronger in case of mild policy intervention, thereby making the policy comparison more complicated.

### The government

The epidemic puts pressure on the government budget, because of a joint erosion of the tax base (less agents work, lower firms profits) and of an increase in transfers (less agents work). I assume that the government balances its budget before the epidemic:

5 $$\begin{aligned} { \tau ^{y} \Pi _{t} + \tau ^{w} w L_{t} = {\bar{G}}+ (1-t) \theta w (N-L_{t}) } \end{aligned}$$

where $L_{t}$ is the total number of workers in period *t*:

6 $$\begin{aligned} { L_{t}=\sum _{j=1}^{J} M_{j t} \, {\mathbbm {1}}_{ [M_{j t} > {\bar{M}}_{j t}] } } \end{aligned}$$

and $\Pi _{t}$ is the sum of all profits:

7 $$\begin{aligned} { \Pi _{t}=\sum _{j=1}^{J} \max \{ (p_{t} y_{j t}- w M_{j t}){\mathbbm {1}}_{ [M_{j t} > {\bar{M}}_{j t}] } \, , \,0 \} } \end{aligned}$$

the quantity ${\bar{G}}$, if positive, is the expenditure that the government finances with the difference between the tax proceedings and the transfers while, if negative, is the extra income needed to finance the expenditures not covered by the proceedings. I assume, differently from Piguillem and Shi (2022), that the tests needed to identify the asymptomatics do not carry any additional cost (I discuss the consequences of costly tests in Sect. 6). I also assume that ${\bar{G}}$ does not change over time, so it is not possible, say, to build additional hospital capacity or to transfer additional resources to the firms.

### Pathogen and timing

Susceptible agents can get the pathogen when matched with an infected. I assume that symptomatic agents transmit the pathogen with probability $\pi$ per match, while asymptomatic agents with probability ${\bar{\pi }}<\pi$. I assume that the contagion probability per single match is independent from previous history, even if, in reality, a repeated exposure to the pathogen increases the probability of infection. Newly infected agents are not infectious for one period.

At the beginning of the period, all agents match with family members, and those who work are matched with all coworkers^2^. They then engage in social activities that result in matches with other members of the economy, say at the supermarket^3^, in the metro, at the restaurant or in schools (I explicitly model schools in Sect. 7). I assume that all agents match with a fixed fraction of the members of the economy who are not in their families or workplaces, but that the identity of those individuals (who exactly each individual meets) changes over time. Importantly, this fraction does not respond to the epidemic progression, meaning that the agents do not voluntarily reduce the number of social interactions to reduce the contagion risk. Once again, this restrictive assumption implies the absence of an endogenous mitigation mechanism.

I assume that a very small fraction of the symptomatics $\psi$ engages in social activities and that their identity changes randomly over time. The idea is that, even if symptomatics are easy to identify and to quarantine, they can still infect others, say the doctor that treats them or a random stranger on the way to the hospital. Asymptomatics, instead, once identified and quarantined, are not allowed to go to work and to have social contacts outside the family until recovery, and they cannot escape the quarantine. At the end of the period, the asymptomatics develop symptoms with exogenous probability $\rho$. I assume that both symptomatics and asymptomatics recover with exogenous probability $\gamma$.

The model features a health system capacity constraint, in line with Favero (2020) and Eichenbaum et al. (2020), among others. If the number of symptomatics patients, at any given time, exceeds the capacity of the health system to properly treat them, the mortality rate increases. More specifically, symptomatic agents die with exogenous probability $\delta$ in case the stock of sympomatics $F_{t}$ is below a threshold, but the death probability grows by a factor $\xi$ above the threshold:

8 $$\begin{aligned} { \delta _{t}=\delta \left[ 1+\xi \, {\mathbbm {1}}_{[F_{t} > g N]} \right] } \end{aligned}$$

where *g* is the indicator of the health system capacity, say the availability of hospital beds.

### Dynamics

The model dynamic is summarized by five equations that describe the evolution of each element of the vector *x*. The first describes the transition to the status of infected and symptomatic:

9 $$\begin{aligned} { f_{\,i j t+1}= (1-\Delta _{t})(1-\Gamma ) f_{\,i j t} + P (1-\Gamma ) a_{\,i j t} } \end{aligned}$$

where $\Delta _{t}$ is a draw from a Bernoulli distribution with parameter equal to the death probability $\delta _{t}$, $\Gamma$ is a draw from a Bernoulli distribution with parameter equal to the recover probability $\gamma$, and where *P* is a draw from a Bernoulli distribution with parameter equal to the probability of developing symptoms $\rho$. Symptomatic agents in *t* are still symptomatic in $t+1$ if they do not recover and if they do not die, while asymptomatics agents become symptomatics if they do not recover and if they develop symptoms. The second equation describes the transition to the status of infected and asymptomatic:

10 $$\begin{aligned} { a_{\, i j t+1}= (1-\Gamma )(1-P) a_{\, i j t} + H_{i j t} s_{\, i j t} } \end{aligned}$$

where $H_{i j t}$ a binary variable equal to one in case of infection in period *t*, defined as follows:

11 $$\begin{aligned} { H_{\ i j t}={\left\{ \begin{array}{ll} 1 &{} \text {prob} \quad 1-\left( 1-\pi \right) ^{{\bar{F}}_{i t}} \left( 1-{\bar{\pi }}\right) ^{{\bar{A}}_{ij t}} \\ 0 &{} \text {otherwise} \end{array}\right. } } \end{aligned}$$

${\bar{F}}_{i t}=F_{\, -i t} +\eta ^{f}_{i t} \, \psi (F_{t}-F_{\, -i t})$ is the number of symptomatics with whom each susceptible is matched, equal to the number of symptomatics in her family $F_{\, -i t}$

12 $$\begin{aligned} { F_{\, -i t}= \sum _{\{{\tilde{i}}\in N(i)\,;\, {\tilde{i}} \ne i\}} f_{\, {\tilde{i}} j t} } \end{aligned}$$

plus a fraction $\eta ^{f}_{i t}$ of all symptomatics who are not in her family and who are able to participate in social activities $\psi (F_{t}-F_{\, -i t})$. The number of asymptomatics with whom she is matched is instead ${\bar{A}}_{i j t}=A_{\, -i t}+A_{\, -j t} +\eta ^{a}_{i t} A_{-i -j t}$, where $A_{\, -i t}$ is the number of asymptomatics in her family and $A_{\, -j t}$ the number of non-quarantined asymptomatics in the firm $m_{j}$ where she works:

13 $$\begin{aligned} { A_{\, -i t}= \sum _{\{{\tilde{i}}\in N(i)\,;\, {\tilde{i}} \ne i\}} a_{\, {\tilde{i}} j t} \qquad \qquad A_{\, -j t}= \sum _{\{{\tilde{j}}\in m_{j}\,;\, {\tilde{j}} \ne j\}} (1-k_{i t}) \, a_{\, i {\tilde{j}} t} } \end{aligned}$$

and where $A_{-i -j t}$ is the number of all non-quarantined asymptomatics who are not in her family or workplace:

14 $$\begin{aligned} { A_{-i -j t}= \sum _{\{j \notin m_{j}\,;\, i \notin N(i)\}} (1-k_{i t}) \, a_{i j t} } \end{aligned}$$

of which only a fraction $\eta ^{a}_{i t}$ is in matchings. The third dynamic equation is for the transition into the status of immune, which is possible only upon recovery (no vaccine is available) and which is absorbing, because the antibodies developed upon recovery protect for life:

15 $$\begin{aligned} { u_{\, i j t+1}= \Gamma f_{\, i j t} + \Gamma a_{\, i j t} + u_{\, i j t} } \end{aligned}$$

The fourth dynamic relationship describes the persistence in the status of susceptible:

16 $$\begin{aligned} { s_{\, i j t+1}=s_{\, i j t} (1-H_{i j t}) } \end{aligned}$$

which happens in case susceptibles do not get the infection. The last dynamic relationship is for deaths:

17 $$\begin{aligned} { d_{\, i j t+1}=\Delta _{t} (1-\Gamma ) f_{\, i j t}+d_{\, i j t} } \end{aligned}$$

which depends on the assumption that only symptomatics who do not recover die. An important statistic to monitor the progression of the epidemic is the effective reproduction number $R_{t}$, equal to the number of new infections in the period per each infected individual: $R_{t}=\frac{1}{F_{t}+A_{t}} \sum _{i=1}^{N} H_{i j t}$. A high effective reproduction number means that pathogen spreads quickly. Containing the epidemic entails bringing the effective reproduction number down to a small level.

## Simulation

The model cannot be solved analytically, so I resort to simulations. I calibrate the model to Italy for a generic pathogen, to make the analysis as general as possible, although some of the parameters will refer to COVID-19. Table 1 list the values used for the baseline simulation, and Sect. 4.1 describes the calibration in great detail. I discuss extensively the robustness to several alternatives in Sect. 7. In “Appendix”, I briefly describe the solution method. In Sect. 4.2, I describe the main policies that I simulate, while, in Sect. 4.3, I introduce the dismal ratios, which I will later use to compare policy outcomes.

**Table 1:** Prameters

Parameter	Description	Value
$\delta$	Death probability	0.025
$\xi$	Death prob multiplier if binding capacity constraint	2
*g*	Health system capacity	0.2
$\rho$	Probability of symptoms	0.25
$\pi$	Contagion probability, symptomatics	0.108
${\bar{\pi }}$	Contagion probability, asymptomatics	0.108
$\gamma$	Recovery probability	0.5
$\eta$	Density of the economy	0.028
$\beta$	Discounting	1
*r*	Interest rate	0
$\theta$	Fraction of the wage to non-workers	0.8
$\psi$	Fraction of symptomatics in social activities	0.1
*K*	Percentage of quarantined asymptomatics	0
$\alpha$	Returns to scale	1
$\lambda$	Probability to reopen a closed firm	0.99
*W*	Labor force participation	0.6
*z*	Fraction of workers below which a firm does not open	0.2
$\tau ^{w}$	Tax rate on labor income and transfers	0.3
$\tau ^{y}$	Tax rate on profits	0.3

### Parameters and calibration

I simulate an economy with $I=500$ families at the weekly frequency. To set the family composition, I use data from the Italian Institute of Statistics (ISTAT). In 2019, 31% of the families were composed by just one member, 27% by two members, 20% by three, 16% by four, and 6% by 5 or more. I cap the last group to 5, and I create families accordingly. The resulting number of agents in the economy *N* is around 1250, which entails an average family size of ${\bar{n}}=N/I \approx 2.5$. Consistently with ISTAT data, I set the employment rate to $W=L/N=0.6$.

According to 2017 ISTAT data, 44% of the workers are employed in firms with 9 employees or less. Therefore I set the fraction of firms with $\vert m_{j} \vert =1$ to 0.44. The number of firms *J*, together with the maximum firm size *O*, are then calibrated so that the total number of workers in non-small firms (size bigger than one) is equal to 0.56%, and such that the average firm size is equal to 3.87 employees, consistently with ISTAT data. The resulting values are $O=12$ employees and $J=64$ firms. This last number implies 1 firm every 7.8 agents, while ISTAT data show 1 firm every 10 individuals. An alternative calibration to match this last value entails a slightly bigger firm size. I analyze the robustness to alternative values in Sect. 7. The lower bound number of workers below which a firm cannot produce is ${\bar{M}}_{j}=\lfloor z M_{j} \rfloor$ with $z=0.2$. The probability $\lambda$ to reopen a closed firm is 0.99 per period. This value implies that 92.3% of the closed firms reopen after a 2-months lockdown, a rather high value that implicitly assumes the existence of generous transfers to firms.

I set $\psi =0.1$, which means that 90% of the symptomatics are randomly excluded from social interactions (the identity of the excluded varies over the simulation runs). In the baseline, no-policy, scenario, I also assume that the asymptomatics are not quarantined, so $\kappa _{i,t}=0 \quad \forall \{i,t\}$.

The total number of per-period matches per individual is $\eta _{i t} N= \eta ^{f}_{it} \psi (F_{t}-F_{-i t})+\eta ^{a}_{it} A_{-i -j t}+\eta ^{s}_{it} S_{-i -j t}+\eta ^{u}_{it} U_{-i -j t}$, where $\eta ^{f}_{it}$ is the fraction of the symptomatics that engages in social activities, $\eta ^{a}_{it}$ is the fraction of the non-quarantined asymptomatics, $\eta ^{s}_{it}$ the fraction of the susceptible who are not in her family or workplace, and $\eta ^{U}_{it}$ the fraction of the immunes who are not in the family or workplace. I assume that, absent a policy intervention, the total number of random matches does not change across individuals (I relax this assumption in Sect. 7) and over time, and I define $\eta _{i t}=\eta$ as the density of the economy. I calibrate the density parameter $\eta$ to have, given the family and firm structure, and given the population size, the average number of contacts per individual used by the the Istituto Superiore di (2020) for the COVID-19 projections in Italy. Averaging their figures over all age classes, with weights equal to the actual population size in 2019 from ISTAT, I obtained 18.5 average contacts per individual. The total number of contacts $SC_{ij}$ is equal to the family size minus 1 plus the number of coworkers plus the number of random matches: $SC_{ij}=\vert n_{i} \vert -1+\vert m_{j}\vert -{\mathbbm {1}}_{\vert m_{j}\vert >0 }+\eta N$. Fixing $(1/N)\sum _{i=1}^{N} SC_{ij}=18.5$, I obtained $\eta =0.011$.

The simulated pathogen is aggressive, in order to have enough variation in the death rate under different policy alternatives and in order for lockdowns and TTQ policies to yield meaningful gains. I assume that the transmission probability is the same for both symptomatics and asymptomatics, and I calibrate it to have a basic reproduction number $R_{0}$ equal to 2, in line with the COVID-19 figures in Chowdhury et al. (2020) and similarly to Influenza and Ebola. Given the 18.5 average contacts per individual, I set $\pi ={\bar{\pi }}=2/18.5=0.1081$.

The baseline death probability is $\delta =0.025$ per week. To make the simulation more realistic, I add an age structure to the model, assuming that a fixed fraction of the population has a 50% higher death probability because of their age, and I set this fraction to 17% according to the population share above 70 in Italy. The multiplier in case of health system stress (binding capacity constraint) is $\xi =2$, similarly to what Eichenbaum et al. (2020a) assume at the peak of the epidemic. I consider a very high benchmark health system capacity, $g=0.2$, although I discuss the results for different capacity levels. I fix the probability to develop symptoms at $\rho =0.25$ and the probability to recover at $\gamma =0.5$. Given this numbers, the average death rate without health system stress is equal to $\delta ^{avg}=0.025 \cdot 0.25 \cdot (0.83+0.17 \cdot 1.5)=0.68\%$, in line with Eichenbaum et al. (2020a) and with the death probability estimated and used by the ISS in Italy for the COVID-19 projections. The probability to remain infected for one period is quite high, $(1-\pi ) \cdot [1-\rho +\rho (1-\delta ^{avg})]=0.676$, which implies a long median infection duration of 6 weeks and a high peak of infections.

The interest rate *r* in the baseline simulation is equal zero and there is no discounting. The agents have a logarithmic utility function. The government transfer $\theta =0.8$ of the wage to pensioners, minors, symptomatics, quarantined asymptomatics and to all workers of the closed firms as an effect of the lockdown. The fixed wage *w* is normalized to 1 without loss of generality. Wages, transfers and profits are all taxed at the 30% rate ($\tau ^{w}=\tau ^{y}=0.3$).

### Policies

I simulate the response to the epidemic conditionally on 3 policies. The first consists of letting the pathogen spread without any intervention. The second is a lockdown that imposes social distancing, and that consists in closing some firms ($M_{k}=0$ for some *k*) and in reducing the social density $\eta$. The third is a TTQ policy with an extensive testing to identify and quarantine the asymptomatics. I assume that all policies have a zero implementation cost (I relax the assumption in Sect. 7), which is a strong assumption for the TTQ.

The lockdown starts 4 weeks after the first infection, runs for a minimum of 8 weeks, and it ends only if the 2-weeks effective reproduction number drops below 0.5. The choice of the starting date is arbitrary, but I discuss the robustness in Sect. 6. The choice of 0.5 as a threshold for the effective reproduction number is arbitrary, although realistic based on what many governments did for the COVID-19 pandemic, and the main results of the analysis proved to be very robust to alternative values between 0.3 and 0.7.

The baseline lockdown consists in closing 50% of the firms and in reducing $\eta$ by three quarters ($\psi /4$ social interactions left). The choice of which firm to close is obviously crucial: for the same proportion of closed firms, there is a milder mitigation in case the closed firms are mostly small. Governments typically choose those firms whose production is not considered essential or strategic, without discontinuing, for instance, the food production and distribution chain. Since there are both big and small firms in those sectors (big supermarkets and corner-shops), I assume that firms are closed at random, but proportionally on size. In greater detail, I set the parameter $\varepsilon$ so that the following expression delivers the target value of 50% closed firms:

18 $$\begin{aligned} { \frac{1}{J} \sum _{l=1}^{{\tilde{l}}} \sum _{j=1}^{J} \quad \lceil \varepsilon {\mathbbm {1}}_{ \vert m_{j} \, \vert =l } \rceil =0.5 } \end{aligned}$$

where ${\tilde{l}}=\max \vert m_{j} \vert$ is the maximum firm size. Then a random fraction of $\varepsilon$ firms of size $\vert m_{j} \vert$ is closed (rounding to the next positive integer). According to this rule, closing 50% of the firms entails having 52% of the workers still at work during the lockdown, which is slightly smaller than the 60% estimated by Barbieri et al. (2020) for Italy. Alvarez et al. (2021) and Farboodi et al. (2021), among others, show that the optimal response to the epidemic involves a slow relaxation of social distancing. I assume instead that all economic and social activities come back to normal as soon as the lockdown ends, which is perhaps a strong assumption given the recent evidence on the post-pandemic mobility rates in Italy (Caselli et al. 2022).

The TTQ policy also starts 4 weeks after the first infection, and it consists of an extensive screening that can successfully and instantaneously isolate a fraction K = 0.5 of the asymptomatics (I discuss the robustness to alternative values in Sect. 6), where:

19 $$\begin{aligned} { K=\frac{\sum _{i=1}^{N} k_{i t} a_{ij t}}{\sum _{i=1}^{N} a_{i t}} } \end{aligned}$$

In all cases, I assume that the agents do not respond to the implemented policies. For instance, workers cannot choose to stay home if their firm is not closed as part of the lockdown.

### Dismal ratios

To summarize the two main trade-offs implied by the policies, I construct two Dismal Ratios. The first is the Output Dismal Ratio (ODR), defined as the percentage of the population spared (if any) for each additional percentage point of output lost:

20 $$\begin{aligned} { ODR=\frac{\frac{1}{N} \left( D_{T}^{nop}-D_{T}^{pol} \right) {\mathbbm {1}}_{[D_{T}^{nop} \, > \, D_{T}^{pol}]}}{\frac{1}{T{\hat{Y}}} \left( \sum _{t=1}^{T} Y_{t}^{nop} - \sum _{t=1}^{T} Y_{t}^{pol} \right) } } \end{aligned}$$

where the superscript *nop* denotes the no-policy scenario, while the superscript $pol \in \{lock;ttq\}$ lockdown or TTQ policies. The second is the Debt Dismal Ratio (DDR), defined as the percentage of the population spared (if any) for each additional unit of debt as a percentage of GDP:

21 $$\begin{aligned} { DDR=\frac{\frac{1}{N} \left( D_{T}^{nop}-D_{T}^{pol} \right) {\mathbbm {1}}_{[D_{T}^{nop} \, > \, D_{T}^{pol}]}}{ B_{T}^{pol} - B_{T}^{nop} } } \end{aligned}$$

where $B_{T}^{nop}$ and $B_{T}^{pol}$ are the debt to GDP ratios at the end o the epidemic (time *T*). A positive dismal ratio means that there is a trade-off: saving lives requires an output reduction or debt increase. A zero dismal ratio means that the policy implies more deaths with respect to non-intervention. A negative dismal ratio means instead that there is no trade-off.

## Simulation results: benchmark

I start each simulation with a *pathogen shock* that consists in the infection of 2 random agents. I simulate the evolution of the epidemic, conditional on the policies, 500 times. I discard the quite rare cases with a limited pathogen circulation and no epidemic, which is possible if early infected agents recover quickly before transmitting the pathogen at a sufficient degree.

In this section I compare policies for the baseline simulation, mostly to illustrate how the model works. In Sect. 6, I summarize the robustness of the results and the policy design, that is all the new insights of my analysis. In appendix, I further analyze the model dynamics with impulse responses to the pathogen shock and with an accounting exercise of new infections.

**Fig. 1:**
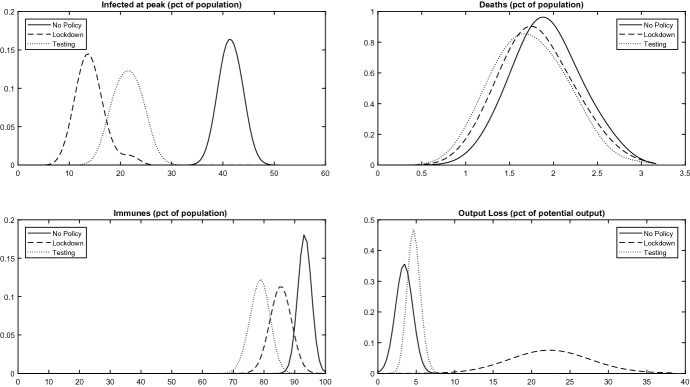
Policy Comparison Notes: Empirical probability density functions of the response to a pathogen shock over simulation runs conditional on three baseline policies. Upper left panel: total number of infected agents (sum of symptomatics and asymptomatics) at the peak of the infection as a percentage of the pre-epidemic population size *N*. Upper right panel: total number of deaths at the end of the epidemic as a percentage of *N*. Lower left panel: total number of immunes at the end of the epidemic as a percentage of *N*. Lower right panel: Cumulative output loss during the epidemic as a percentage of potential GDP

Figure 1 plots the empirical distributions, over simulation runs, of 4 key policy performance indicators: the percentage of infected at peak, the death rate at the end of the epidemic, the percentage of immunes at the end of the epidemic and the cumulative output loss (as a percentage of potential output). The summary statistics are summarized in Table 2, alongside two additional variables: the debt to GDP ratio at the end of the epidemic and inflation (percentage difference between the price at the end of the epidemic and the price at the beginning). Square brackets, in what follows, indicate 95% confidence intervals.

The upshot of this section is that lockdowns yield, on average, a mortality reduction at a high cost in terms of foregone output and increased debt, although the death rate with a lockdown is not smaller than in the no-policy benchmark in many simulations. TTQ policies, on the other hand, yield better outcomes. Both mitigation policies, however, are associated to a significant volatility of outcomes.

Lockdowns make epidemics last longer: a median of 37 weeks [32; 47] to reach zero infected in the economy, as compared to the 22 [20; 27] weeks of the no-policy alternative and to the 28 [23; 35] of the TTQ. The median peak of infection is 13.8% [10%;  19.5%] with a lockdown, as compared to 41.8% [38.9%;  44.5%] in the no-policy scenario and to 21.3% [17.6%;  25.5%] of the TTQ. All peaks are actually quite high, because of the 67.6% probability to remain infected in the benchmark simulation. Lockdowns reduce the total number of infections over the course of the epidemic, resulting in a lower fraction of immunes: a median of 85.3% [80.9%;  88.5%] versus 93.3% [91.7%;  94.5%] in the no-policy scenario. The median death rate with a lockdown is 1.84% [1.22%;  2.47%], lower than the 1.95% [1.38%;  2.68%] of the no-policy scenario. The TTQ policy, by reducing contagions from asymptomatics, also allows for a considerable reduction of infections, with 78.5% [74.4%;  81.8%] median immunes at the end of the epidemic and a median 1.71% [1.13%;  2.37%] death rate.

An important feature of the simulations and, in general, of my analysis, is pointing out that the standard deviation of the death rate is quite high in all scenarios, resulting in large confidence intervals. In other words, lockdowns and TTQ policies do not necessarily reduce the number of deaths with respect to the no-policy alternative. For instance, they might result in more deaths if, among the lower number of infected, there is a higher share of high-risk individuals, which is more likely in case they live in big families or work in big firms. Similarly, if there are a lot of asymptomatics working in big firms before a lockdown, there will be many infection and deaths regardless of it.

Lockdown are costly, both for the current and for the future generations. The median output loss induced by a lockdown is in fact 22.1% [17.4%;  28.7%], almost 7 times as big as the median loss in the no-policy scenario, and almost 5 times as big as the loss associated to the TTQ policy (assuming that tracing and testing asymptomatics is costless). The median debt to GDP ratio increase at the end of the epidemic, which is a measure of the cost for future generations, is instead equal to 15.3% [10.7%;  22.5%] in case of a lockdown, ten times as big as in the no policy scenario and seven times as big as in the TTQ scenario.

**Fig. 2:**
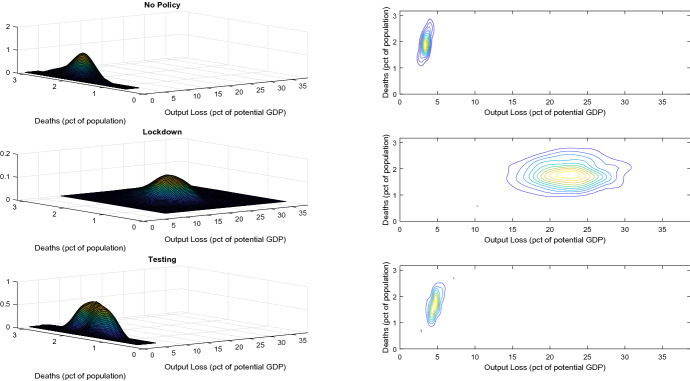
Output Loss and Mortality. mpirical probability density functions of the simulation results for the three baseline policies (see text for details): no policy (solid line), lockdown (dashed line) and TTQ (testing, dotted line).

Figure 2 shows the joint distribution of the death rate and of the output loss in the three policy scenarios. Clearly the output loss determined by a lockdown is very high and variable, while mortality is not necessarily smaller.

The first three rows of Table 3 report the medians of the dismal rations over the simulation runs. The last column of Table 3 reports a crucial information to evaluate a policy: the percentage of zero dismal ratios. The median ODR of a lockdown is 0.0049 [0;  0.0556]. For a population of 60 millions, this means that a lockdown saves a median of 2940 lives for each unit of output lost. The median DDR of a lockdown is instead 0.0067 [0;  0.0752], which implies 4020 lives saved for each additional percentage point of the debt to GDP ratio. The problem is that a lockdown does not imply a lower number of deaths in 46% of the simulations. The median ODR of the TTQ policy is 0.2112 [0;  2.0245], while the median DDR 0.4074 [0;  3.5938]. The median number of lives saved by the TTQ policy are thus 126720 for each unit of output lost and 244440 for each additional unit of debt. TTQ policies do not reduce the mortality in 34% of the cases, which is less than in lockdown scenario.

## Simulation results: robustness and policy design

The novel feature of my analysis is that rich model structure allows the study of the factors that influence the performance of the mitigation policies. I performed several alternative exercises, and the results are summarized in Tables 3 and 4. In short, I find that lockdowns work best in case of an intermediate health system capacity, in case of high mortality rates, with very contagious pathogens and in case of a small recovery probability. I also find that lockdowns are more effective with older agents, smaller families, bigger firms, dense societies and with a more diffused labor market participation. Lockdowns are best if prolonged until the reproduction number reaches a small value and if implemented soon. In all cases, however the ODR and DDR of the lockdown are below the ones of the TTQ policy, unless in case of limited TTQ policies that are not able to identify a big share of the asymptomatics.

**Fig. 3:**
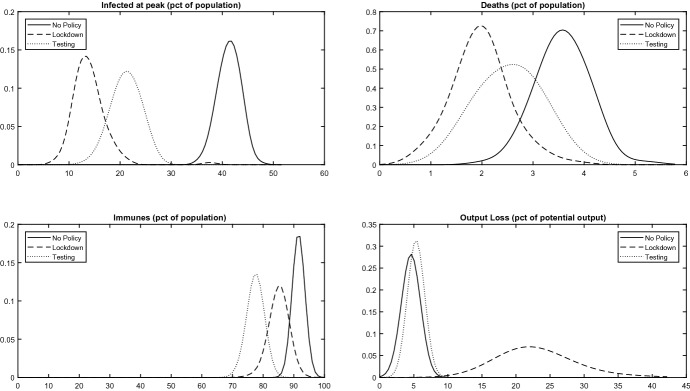
Policy Comparison. Binding Health System Capacity Constraint. Empirical probability density functions of the response to a pathogen shock over simulation runs conditional on three baseline policies with binding health system capacity constraint $g=0.05$. Upper left panel: total number of infected agents (sum of symptomatics and asymptomatics) at the peak of the infection as a percentage of the pre-epidemic population size *N*. Upper right panel: total number of deaths at the end of the epidemic as a percentage of *N*. Lower left panel: total number of immunes at the end of the epidemic as a percentage of *N*. Lower right panel: Cumulative output loss during the epidemic as a percentage of potential GDP.

*Health System Capacity* In case of a smaller health system capacity than benchmark, lockdowns and TTQ policies, by smoothing the peak of infections, save many more lives, but TTQ policies are still associated with sharper gains. Figure 3 shows the densities of the outcomes in case of $g=0.05$. In case of a very small health system capacity, however, the constraint binds even in case of lockdowns and TTQ policies, so the gains from both policies are smaller. In case of a very big capacity, conversely, the constraint never binds, so the gains are also smaller. Thus policy interventions are best in case of intermediate health system capacities.

*Pathogen Characteristics* The gains of a lockdown increase with the mortality rate and with the contagiousness of the pathogen, but they decrease with the recovery probability. TTQ policies are less effective in case of very contagious diseases, or if the infected are slow to recover. In all cases, however, the ODR and DDR of the TTQ policy are above the ones of a lockdown.

*Country Characteristics* In case of a higher density, which is more representative of large and densely populated metropolitan areas, contagions are much faster and, without any policy intervention, the mortality, the output loss and the debt increase are also steeper. Lockdowns are more effective in this scenario, while the benchmark TTQ policy is less effective because stemming the pathogen diffusion requires the identification of a bigger faction of the asymptomatics. With smaller families, lockdowns are more effective, because contagions within the family become a less important source of contagion at the country level, and because contagions within the family are not affected by a lockdown. Conversely, TTQ policies are less effective with smaller families, since contagions from social activities become relatively more important. Similarly, lockdowns and TTQ policies are more effective in case of bigger firms and in case of a more diffused labor market participation, because they significantly reduce infections on the job. The gains from lockdowns and TTQ policies are also higher in case of an older population since, for the same output loss and debt increase, there is a steeper mortality decrease.

*Lockdown Design* A more severe lockdown, that forces more firms to close, implies a higher median output loss and a higher debt increase. The effective reproduction number drops below threshold quickly, but this means opening the economy with few immunes, and infections raise soon after the lockdown ends, with a resulting median death rate that turned out to be higher than benchmark. Discontinuing only the social interactions reduces the output loss and the debt increase of a lockdown, and the median mortality rate is also lower in this case. At the opposite extreme, shutting down firms, while keeping the same social density, results in a median death rate which is not very different from the no-policy benchmark, and in a much higher output loss and debt increase. These results are the consequence of the high number of small firms and of the prominence of social contacts as a source of infections. Following Favero (2020), I also considered an alternative approach to the lockdown, preventing high-risk individuals only from going to work. Since the fraction of high risk individuals in the baseline simulation is rather small, and since some of those are pensioners (not in the labor force), I ended up with results in line with no-policy scenario.

A more stringent stopping rule, with a 0.25 threshold, delivers longer lockdowns and longer lasting epidemics, resulting in sharper output losses and debt increases, but also in smaller death rates. Conversely, a less stringent stopping rule, with a 0.75 threshold, delivers average results in line with the benchmark, although more volatile. The conclusion is that it is better to re-open the economy when the effective reproduction number is sufficiently low, although this means prolonging the lockdown and the epidemic. Taking the reasoning to the extreme, a zero threshold results in a very high median epidemic duration, ans in quite big output losses and debt increases, although the mortality rate drops considerably. The problem with such extreme lockdowns, however, is that they might be hard to enforce. Starting the lockdown earlier results in a lower mortality, but in longer lockdowns and longer epidemics, with a more pronounced output loss and debt increase. Later starts, conversely, deliver results in line with the benchmark.

*TTQ Design* Isolating only 25% of the asymptomatics results in a higher peak of infections and in a higher median death rate, and many simulations do not actually deliver gains. Conversely, isolating 75% of the asymptomatics implies a sharp decrease of the death rate and a much smaller output loss, resulting in very high ODR and DDR, with no gains in few simulations. Thus the TTQ policy, to be effective, requires the identification of a big fraction of the asymptomatics. To achieve that, it is necessary to have a high, perhaps unrealistic, testing capacity, or to adopt more sophisticated testing strategies (see Russo 2022 for an analysis).

## Extensions

In Sect. 7.1 I study the effects of second waves of infections. In Sect. 7.2, I extend the model to formally include schools. In Sect. 7.3, I extend the model to a two-countries setting. In Sect. 7.4, I consider a model with heterogeneous social participation and with firms heterogeneity over the social contacts required for production. In Sect. 7.5, I add a test cost to the model.

### Second waves

To evaluate the vulnerability to a second wave of infections, I performed the following exercise: for all simulation runs, and conditional on the benchmark policies, I infected two randomly chosen agents who, at the end of the epidemic, were still susceptible. Repeating this experiment 100 times, I then computed the percentage of simulations that resulted in a number of infected bigger than 6 (three times the stocks of new infected) four periods after this second shock. The median percentage of such second waves was zero for all three policies. In case of a more severe lockdown, with a 0.1 threshold, I also obtained a zero median percentage of second waves. The risk of second waves is indeed remote.

### Schools

In the baseline simulation, schools were bunched with other social activities. I extended the model assuming that a fixed fraction of the agents who do not work, and who live in families of three or more^4^, attends a school. This fraction is in turn calibrated to match the percentage of the Italian total population that attends daycare, schools and universities. According to ISTAT data, in Italy 38.5% of the agents aged between 19 and 25 attend a university and 25% of the kids aged less than 3 go to daycare. Assuming that all kids aged between 3 and 18 attend a school, I obtain a total fraction of 18%. I set the number of schools in the model in order to have the average school size from ISTAT. I also re-calibrated the parameter $\eta$ to have the average target number of contacts per individual, including schools, of 18.5. In all simulations, symptomatics and quarantined asymptomatics are not allowed to attend. Assuming that all schools are closed during a lockdown, I obtained almost identical results to the benchmark. Closing schools only does not have an effect on production, but it also fails to slowing down contagions, resulting in a modest reduction of the death rate and in zero median ODR and DDR. Those simulations that resulted in a lower mortality rate, however, resulted in negative ODR and DDR, given the zero short-run cost (The long-run cost of closing schools and universities cannot be properly evaluated in the model).

### Two countries

To assess the importance of external links, and to evaluate the effect of closing the borders to travel, I extended the model to a two-countries (or regions) setting. In this economy, social activities entails also matches with agents from the foreign country, although not for symptomatics and quarantined asymptomatics. I set the intensity of those matches, or the percentage of the foreign population with whom each resident is matched, to ${\bar{\eta }}=\eta / 2$, and I assumed identical economies. The simulation works as follows: the initial pathogen shock hits the foreign country and it is then imported in the home country via social contacts. Four weeks after the beginning of the epidemic (in the foreign country), both the home and the foreign country implement the same policy. During the lockdown, ${\bar{\eta }}$ is zero. Absent a re-calibration of $\eta$, there is a smaller median duration of the epidemic and a higher median percentage of infected at peak in all policy scenarios, with a higher death rate and a higher output loss. Lockdowns are more effective, since they shutdown an important source of contagions, with ODR and DDR in the home country equal, respectively, to 0.0111 and 0.0168 (zeros in 43% of the simulations). TTQ policies with open borders are instead less effective. Closing the borders only, while keeping social and economic activities unchanged, has the advantage of reducing contagions without any output costs, and results in a negative median ODR. The conclusion is that closing borders must be a priority. The model can be further generalized to more than two heterogeneous countries and regions, to evaluate the opportunity of closing only a subset of all borders, but such exercises are beyond the scope of this work.

### Heterogeneous social matchings

I extended the model to account for heterogeneous participation in social activities, relaxing the assumption of $\eta _{it}=\eta \quad \forall \{i,t\}$. More specifically, while still keeping the time invariance assumption, I modeled a Gamma distribution for the social contacts, with parameters calibrated in order to match the mean and variance of the distribution of social contacts, respectively 18.5 and 26. The results are almost indistinguishable from the baseline. A selective lockdown for the highly-social individuals, regardless of their infections status, wold be very effective at stemming the epidemic, but it would be difficult to legally enforce.

One of the simplifying assumptions of the benchmark model specification is that agents meet, each period, with all coworkers, regardless of the firm size. The problem is that there might be firms where production requires a bigger number of social contacts to be carried out, and this heterogeneity might be important both for the pathogen diffusion and for the effectiveness of the mitigation policies. In practice, there might be big firms where workers seldom meet and small firms where production is characterized by frequent interaction, meaning that the number of coworkers in matchings is not necessarily proportional to the firm size. I extended the model assuming that working agents in firms with size bigger than one match, every period, just with a fraction of their coworkers, and I draw this fraction, at the firm level, from a uniform distribution between zero and one. With this additional assumption, workers in smaller firms can also have more contacts, on the workplace, than workers in bigger firms. The results of the simulations turned out to be in line with the benchmark. In this alternative model, it is also possible to study an additional approach to lockdowns, closing only the firms whose production requires a bigger number of social interactions. The results are reported in Table 4 for the case of a lockdown that forces to close all firms, of size bigger than one, where each worker meets with more than 50% of its coworkers per period, and where firms of size one are closed with a 50% probability (for consistency). The results are not different from the ones obtained by the benchmark lockdown policy with random firms closures. In conclusion, differences in the organization of work do not seem relevant drivers of the simulation results.

### Test cost

It is possible that TTQ policy is preferred to a lockdown because the tests themselves do not carry any cost. To check for robustness, suppose that a single test costs 10 euros and, rather unrealistically, that the cost is the same regardless of how many tests are processed. Suppose also that the tests are deficit financed, so the ODR computations do not change. Yearly GDP per capita in Italy is roughly 34500 euros, so 10 euros are equivalent to 1.5% of the weekly GDP per capita. Suppose that, in order to discover the target 50% of all asymptomatics, the government needs to administer tests to 10% of the population every period. The total cost amounts to 0.15% of nominal GDP which, for 20 weeks, is 3% of weekly GDP. The median debt to GDP ratio at the end of the epidemic is 2%. Thus the correction factor to have a DDR that includes the tests cost is 2/5. In the baseline simulation, the median DDR of the TTQ policy is 0.4074. Multiplying it by 2/5 gives 0.1629 which is still far above the DDR of the lockdown. Even assuming that a test costs 50 euros, with a correction factor of 2/17, results in a DDR of 0.047.

## Conclusion

Coping with an epidemic asks for difficult calls. Social distancing slows down the epidemic and can save lives, but at the cost of discontinuing social and economic activities, with an increase of public debt. My simulation results show that lockdowns do indeed save lives on average, although not always, but at a high cost for the current and future generations. Lockdowns are more effective with older agents, smaller families, bigger firms, dense societies and diffused labor market participation. TTQ policies, in general, yield better results but, in order to work properly, they must involve a through testing of the population which is not always feasible.

Lockdowns involve a delicate balance of interests, first, and foremost, regarding who will bear their costs. As such, they involve complicated political negotiations, perhaps also resulting in a war-of-attrition type of situation. However there is an important political advantage associated with them, which is among the reasons why they were so popular in response to the COVID-19 outbreak: with a high death rate despite of a lockdown, it is still possible to claim that there could have been more deaths without; in case of few deaths, it is even easier to claim a success. The Italian case is a good example of a government that gained consensus during a deadly COVID-19 outbreak, while the widespread criticism to the essentially non-interventionist strategy of the Swedish government shows the opposite side. A complete comparative evaluation of alternative policies cannot abstract from the their political convenience. I leave such an analysis to future work.

## Figures and Tables

**Fig. 4:**
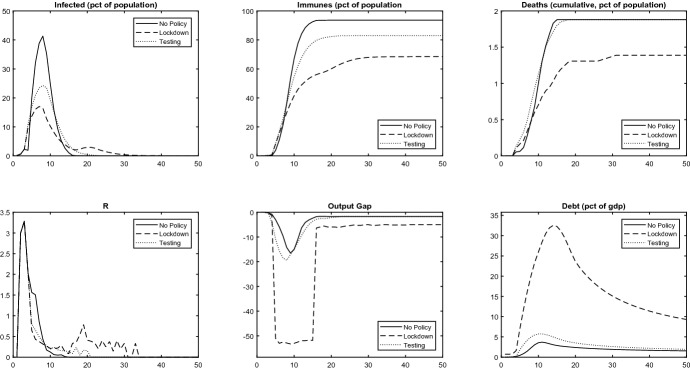
Impulse Response to the Pathogen Shock. Example 1. Impulse response to the pathogen shock consisting of two randomly infected agents. Upper left panel: total number of infected agents (symptomatics plus asymptomatics) as a percentage of the pre-epidemic population size *N*. Upper central panel: total number of immunes as a percentage of *N*. Upper right panel: total number of deaths as a percentage of N. Lower left panel: 1-week effective reproduction number. Lower central panel: output gap. Lower right panel: Debt to GDP ratio, where the debt at time *t* (sum of all budget deficits or surpluses from 0 to *t*.)

**Fig. 5:**
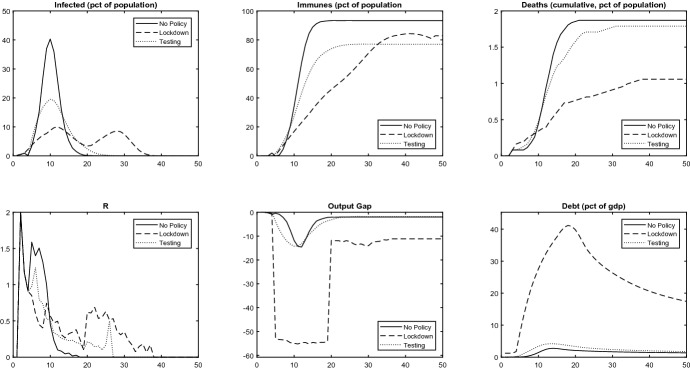
Impulse Response to the Pathogen Shock. Example 2. Impulse response to the pathogen shock consisting of two randomly infected agents. Upper left panel: total number of infected agents (symptomatics plus asymptomatics) as a percentage of the pre-epidemic population size *N*. Upper central panel: total number of immunes as a percentage of *N*. Upper right panel: total number of deaths as a percentage of N. Lower left panel: 1-week effective reproduction number. Lower central panel: output gap. Lower right panel: Debt to GDP ratio, where the debt at time *t* (sum of all budget deficits or surpluses from 0 to *t*.)

**Fig. 6:**
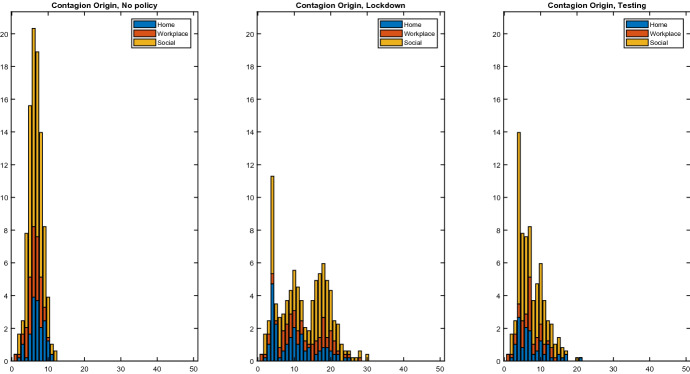
Breakdown of New Contagions, Example. Breakdown of new contagions, in percentage of the population, due to family, workplace and social contacts conditional on the three baseline policies (see text for details): no policy (left panel), lockdown (central panel) and TTQ (right panel)

## Data Availability

All data used for the simulations are publicly available.

## Appendix 1: Model Solution Algorithm

The model is solved on a discrete grid. All agents start as susceptible. Then random infections hit. The status at the end of the period is computed according to Eqs. 9, 10, 15, 17 and 16. Production is determined by the number of available workers at the beginning of the period. Income is also determined by the status at the beginning of the period. Consumption is chosen optimally given the realized income stream over the simulation period and given the interest rate, the discount rate and the utility function. Government debt is simply computed according to Eq. 5, given per-period production and given the optimal consumption/saving choices. During a lockdown, a fixed fraction of the firms is closed and a fixed fraction of social contacts is discontinued, but the solution proceeds in the same way. With a TTQ policy, the solution algorithm is also unchanged except for the fixed fraction of asymptomatics that does not engage in social activities.

### Appendix 2: Impulse Responses

Figure 4 plots one of the possible impulse responses to the initial pathogen shock under the three baseline policies. The epidemic unfolds rather quickly, and it lasts longer in case of a lockdown. The lockdown is effective at smoothing the peak of infections. There is a slight increase in contagions as soon as the lockdown ends, which however fades away quite rapidly. The TTQ policy results are in between. The percentage of immunes is highest in the no-policy scenario and smallest in case of a lockdown, while deaths are lowest with a lockdown. The output gap drops considerably when the lockdown starts, but there is a sensible output loss even in the no-policy and TTQ scenarios, since the symptomatics (in the no-policy case) and the quarantined asymptomatics (in the TTQ case) cannot work. After the epidemic, the output gap is very close to zero in the no policy and TTQ scenarios, and smallest in case of a lockdown. Debt also increases quickly with a lockdown, because of the lower tax proceedings and of the higher transfers to the unemployed. It reverts as soon as the lockdown ends, although, by the end of the epidemic, it is still above the level obtained in the no-policy and TTQ scenarios.

The impulse responses in Fig. 4 refer to just one of the possible responses of the economy. For instance, contagions might increase a lot when the lockdown ends, with a second peak of infections. This is likely to happen when the lockdown is very fast at reducing the effective reproduction number, leaving only few immunes. Figure 5 plots the impulse responses for such a case. In general, the results obtained under alternative policies are contingent on the identity of the agents that are infected at each stage of the epidemic. I study extensively this variability in the next section.

### Appendix 3: Infections Breakdown

To better understand how the policies work, Fig. 6 plots, for one of the possible impulse responses, the breakdown of new infections due to contacts within the family, at the workplace and due to social activities. Since the firms are, on average, small (many of them have just one worker), and since the labor market participation is quite low in this calibrated economy, workplace contacts account, on average, for only 20% of all contagions^5^ in the no-policy scenario. Most of the contagions, around 60% on average, are instead due to social contacts, and the remaining 20% to contacts within the family. Lockdowns and TTQ policies reduce the total number of contagions and increase the relative importance of contagions within the family.

**Table 2:** Policy comparison

	No policy				Lockdown				TTQ			
	Med	Std	95% conf int		Med	Std	95% conf int		Med	Std	95% conf int	
Duration	22	2.077	20	27	37	4.734	32	47	28	3.939	23	35
Infpeak	41.792	1.759	38.916	44.498	13.854	3.131	10.041	19.506	21.283	2.456	17.590	25.513
Deaths	1.954	0.389	1.382	2.683	1.841	0.398	1.221	2.469	1.709	0.380	1.134	2.369
Immunes	93.301	0.827	91.667	94.490	85.322	2.379	80.991	88.525	78.457	2.245	74.424	81.811
YLoss	3.486	0.464	2.712	4.287	22.156	3.521	17.414	28.729	4.700	0.577	3.837	5.651
Debt	1.497	0.165	1.223	1.769	15.308	3.679	10.724	22.538	2.098	0.244	1.708	2.504
Inflation	2.115	0.542	1.203	3.087	7.794	2.821	3.841	13.049	1.873	0.500	1.187	2.714

Duration is the length of the epidemic defined as the first period with zero infected. Infpeak is the maximum percentage of infected agents (sum of symptomatics and asymptomatics) in a given period during the epidemic. Deaths is the total number of deaths at the end of the epidemic as a percentage of the pre-epidemic population size. Immunes is the total number of immunes at the end of the epidemic as a percentage of the pre-epidemic population size. YLoss is the cumulative output loss over the epidemic as a percentage of potential GDP in the same time span. Debt is the Debt to GDP ratio at the end of the epidemic. Inflation is the percentage difference between the price at the end of the epidemic and the price at the beginning. Med stands for median over all simulation runs. std stands for standard deviation over all simulation runs. 95% conf int is the 95% confidence interval over all simulation run (5% of the simulations resulted in a lower value than the lower bound and 5% in a bigger value than the upper bound)

**Table 3:** Policy comparison, robustness

	Policy	Duration	Deaths	YLoss	Debt	ODR	DDR	No gain
Baseline	No Policy	22	1.9544	3.4857	1.4972			
	Lockdown	37	1.8410	22.1561	15.3078	0.0049	0.0067	45.7
	Testing	28	1.7087	4.7005	2.0975	0.2112	0.4074	34.0
Highdens	No Policy	19	2.1053	4.2025	1.8604			
	Lockdown	30	1.8791	20.0272	13.0270	0.0170	0.0226	36.8
	Testing	20	1.9721	6.3152	2.9321	0.0920	0.1594	37.9
Smallfam	No Policy	21	2.0257	3.7689	1.6688			
	Lockdown	34	1.8798	20.9140	13.8709	0.0105	0.0158	39.8
	Testing	25	1.8206	5.5130	2.5358	0.0847	0.1607	39.8
Bigfirms	No Policy	23	1.9624	3.3850	1.4360			
	Lockdown	40	1.8669	22.3905	15.9534	0.0084	0.0114	37.5
	Testing	30	1.5523	4.3092	1.8998	0.4824	0.9757	22.5
Lab75	No Policy	21	2.0375	3.7024	1.4600			
	Lockdown	34	1.7843	21.1242	14.3892	0.0129	0.0159	42.3
	Testing	25	1.7915	5.1972	2.3338	0.1061	0.2387	35.1
Older	No Policy	22	2.1259	3.5775	1.5112			
	Lockdown	38	1.8791	22.2619	14.9563	0.0098	0.0132	40.7
	Testing	28	1.8730	4.9107	2.0942	0.1102	0.3591	39.5

Duration is the length of the epidemic defined as the first period with zero infected. Deaths is the total number of deaths at the end of the epidemic as a percentage of the pre-epidemic population size. YLoss is the cumulative output loss over the epidemic as a percentage of potential GDP in the same time span. Debt is the Debt to GDP ratio at the end of the epidemic. ODR is the Output Dismal Ratio defined in Eq. 20. DDR is the Debt Dismal Ratio defined in Eq. 21. The table reports the median outcomes over all simulation runs conditional on three policy approaches, no policy, lockdown and testing, for different scenarios (first column). No gain pct is the percentage of simulation runs with zero ODR and DDR, so with lockdowns or testing strategies resulting in a higher death rate with respect to the no-policy alternative. The Scenarios are the following: Baseline is the benchmark simulation; Highdens entails doubling the density $\eta$; Smallfam entails a 50% share of singles; Bigfirms entail halving the share of firms with 1 employee; Lab75 entails $W=0.75$ or 75% labor force participation; Older entails doubling fraction of the population with higher death rate upon infection

**Table 4:** Lockdown design and testing efficacy

	Duration	Mortality	YLoss	Debt	ODR	DDR	No Gain
No policy	22	1.9544	3.4857	1.4972			
Lockdown, bench	37	1.8410	22.1561	15.3078	0.0049	0.0067	45.7
Lockdown, thresh 0.75	33	1.8914	16.2127	9.7426	0.0065	0.0091	46.7
Lockdown, thresh 0.25	49	1.7087	26.4205	19.9464	0.0117	0.0128	30.7
Lockdown, thresh 0	54	1.4646	31.4121	25.7163	0.0221	0.0223	22.3
Lockdown, firms mild	37	1.7129	13.2070	7.4737	0.0339	0.0549	37.6
Lockdown, firms severe	37	1.9704	29.2497	22.5149	0.0000	0.0000	54.1
Lockdown, social mild	32	1.8745	19.6246	12.6348	0.0088	0.0119	46.1
Lockdown, social severe	36	1.8868	21.6179	14.7671	0.0000	0.0000	50.6
Lockdown, firms only	23	2.0458	18.0390	10.9092	0.0000	0.0000	57.7
Lockdown, social only	35	1.7213	3.0543	1.1134	− 0.3859	− 0.4073	37.1
Lockdown, early	43	1.7974	26.0673	19.4113	0.0090	0.0108	40.5
Lockdown, late	45	1.8714	25.0393	18.1755	0.0063	0.0077	39.3
Lockdown, socprod	38	1.9769	21.5888	14.8263	0.0037	0.0047	46.8
TTQ, bench	28	1.7087	4.7005	2.0975	0.2112	0.4074	34.1
TTQ, 0.25	23	1.8946	4.4000	1.9900	0.0000	0.0000	55.6
TTQ, 0.75	37	1.1410	3.9357	1.6076	0.5617	0.8903	4.9

Duration is the length of the epidemic defined as the first period with zero infected. Deaths is the total number of deaths at the end of the epidemic as a percentage of the pre-epidemic population size. YLoss is the cumulative output loss over the epidemic as a percentage of potential GDP in the same time span. Debt is the Debt to GDP ratio at the end of the epidemic. ODR is the Output Dismal Ratio defined in Eq. 20. DDR is the Debt Dismal Ratio defined in Eq. 21. The table reports the median outcomes over all simulation runs conditional on policy (first column). No gain pct is the percentage of simulation runs with zero ODR and DDR, so with lockdowns or testing strategies resulting in a higher death rate with respect to the no-policy alternative. Policies: No policy means no policy intervention; Lockdown, bench is the baseline lockdown with 8 weeks minimum requirement, 50% of firms closed, 75% of social activities discontinued and end when the 2-weeks effective reproduction number drops below 0.5; Lockdown, thresh 0.75, Lockdown, thresh 0.25 and Lockdown, thresh 0 are lockdowns that end when the 2-weeks effective reproduction number drops below 0.75, 0.25 or at 0. is a lockdown that ends when the 2-weeks effective reproduction number drops below 0.25; Lockdown, firms mild and Lockdown, firms severe are lockdowns that close 25% and 75% of the firms; Lockdown, social mild and Lockdown, social severe are lockdowns that discontinue 50% and 87.5% of the social activities; Lockdown, firms only is a lockdown that does not discontinue social activities; Lockdown, social only is a lockdown that does not close firms; Lockdown, early and Lockdown, late start the third and sixth week after the pathogen shock; Lockdown, socprod refers to lockdown that force to close only the firms with size bigger than one where each worker meets with more than 50% of the coworkers per period and where size one firms are closed with 50% probability. TTQ, bench, TTQ, 0.25 and TTQ, 0.75 are testing strategies that are able to isolate and quarantine 50%, 25% and 75% of the asymptomatics every period

## Footnotes

1 In this paper I will use COVID-19 to denote both the pathogen and the disease that it induces, although the true pathogen name is SARS-Cov-2.

2 I assume that the contagion probability on the job does not depend on occupation and on the firm characteristics, even if there is evidence of variation (Caselli et al. 2021).

3 Consumption activities are not separate contagion opportunities from social interactions. This excludes demand effects that exacerbate the recessionary effect of an epidemic as in Eichenbaum et al. (2020a): the agents simply decide to decrease consumption to limit their exposure to the pathogen.

4 I assume that single or divorced parents either live with someone else, like a grandparent, or that the divorced parents, which are allowed to see the kids, make up for a single, extended, family unit.

## Code availability

All codes for replication will be made available upon request.
